# High-Throughput System for the Presentation of Secreted and Surface-Exposed Proteins from Gram-Positive Bacteria in Functional Metagenomics Studies

**DOI:** 10.1371/journal.pone.0065956

**Published:** 2013-06-14

**Authors:** Dragana Dobrijevic, Gaetana Di Liberto, Kosei Tanaka, Tomas de Wouters, Rozenn Dervyn, Samira Boudebbouze, Johan Binesse, Hervé M. Blottière, Alexandre Jamet, Emmanuelle Maguin, Maarten van de Guchte

**Affiliations:** 1 INRA, UMR1319 Micalis, Jouy-en-Josas, France; 2 AgroParisTech, UMR Micalis, Jouy-en-Josas, France; Charité-University Medicine Berlin, Germany

## Abstract

Complex microbial ecosystems are increasingly studied through the use of metagenomics approaches. Overwhelming amounts of DNA sequence data are generated to describe the ecosystems, and allow to search for correlations between gene occurrence and clinical (e.g. in studies of the gut microbiota), physico-chemical (e.g. in studies of soil or water environments), or other parameters. Observed correlations can then be used to formulate hypotheses concerning microbial gene functions in relation to the ecosystem studied. In this context, functional metagenomics studies aim to validate these hypotheses and to explore the mechanisms involved. One possible approach is to PCR amplify or chemically synthesize genes of interest and to express them in a suitable host in order to study their function. For bacterial genes, *Escherichia coli* is often used as the expression host but, depending on the origin and nature of the genes of interest and the test system used to evaluate their putative function, other expression systems may be preferable. In this study, we developed a system to evaluate the role of secreted and surface-exposed proteins from Gram-positive bacteria in the human gut microbiota in immune modulation. We chose to use a Gram-positive host bacterium, *Bacillus subtilis*, and modified it to provide an expression background that behaves neutral in a cell-based immune modulation assay, *in vitro*. We also adapted an *E. coli – B. subtilis* shuttle expression vector for use with the Gateway high-throughput cloning system. Finally, we demonstrate the functionality of this host-vector system through the cloning and expression of a flagellin-coding sequence, and show that the expression-clone elicits an inflammatory response in a human intestinal epithelial cell line. The expression host can easily be adapted to assure neutrality in other assay systems, allowing the use of the presented presentation system in functional metagenomics of the gut and other ecosystems.

## Introduction

Metagenomics has transformed modern microbiology, allowing us to study microorganisms that have been refractory to cultivation in the laboratory. Through the use of metagenomic approaches the amount of new information regarding complex ecosystems is rapidly increasing, as exemplified by the recent publication of a catalog of 3.3 million bacterial genes from the human gut microbiota [1]. In parallel, there are strong indications that the gut microbiota plays a pivotal role in human health, going far beyond its accepted function in the digestion and energy harvesting of alimentary components. Correlation studies of gut microbiota composition and disease parameters strongly suggest a role of the microbiota in (the prevention of) inflammatory bowel diseases, obesity and other diseases [2]–[5], while experiments in mice indicate a role in hitherto unsuspected processes like the maturation and modulation of the immune system [6]–[8] and even in behavior [9].

The next challenge is to move from correlations to functional relationships, i.e. to the identification of the bacterial effectors responsible for the observed effects, and the description of the mechanisms involved. Important progress has been made in mice experiments, where especially the establishment of germ-free and genetically modified mice have been major breakthroughs, allowing to test the effects of specific bacterial populations and to get a hold on their mode of action. To predict effects in humans, human cell cultures have been instrumental, notably in the field of immune modulation. Cell-based assays not only permit to test the effects of bacteria within the context of living human cells, but also to study the underlying cellular mechanisms. Importantly, they constitute a powerful and straightforward approach allowing the parallelized functional screening of high numbers of bacterial strains [10]–[12].

In the context of metagenomic studies, individual bacterial strains are usually not available because they never have been, or even could be, isolated and cultured in the laboratory. An approach to circumvent this problem consists of the generation of metagenomic libraries with large DNA inserts in *Escherichia coli* (e.g. [13], [14]). Alternatively, in a directed approach genes of potential interest may be identified by *in silico* analyses of metagenomic data, amplified by PCR or chemically synthesized, and expressed in host bacteria which can then be brought into contact with human cell cultures. Usually *E. coli,* for which high-throughput cloning systems like the Gateway system for site specific recombination based cloning [15] have been developed, is used as the heterologous expression host.

Genes of potential interest identified in metagenomic data may encode surface proteins or secreted proteins from Gram-positive bacteria, however, which constitute clear examples of proteins where a Gram-positive expression host would be preferred over *E. coli* with its Gram-negative cell envelope architecture [16]. In the case of the study of immune modulation properties of the human GI tract microbiota this is an important consideration, as Gram-positive bacteria make up roughly 50% of the GI tract microbiota [17], and a growing number of studies on probiotic bacteria attribute immune-modulation activity to surface associated and extracellular proteins [18].

*Bacillus subtilis*, a widely studied Gram-positive model bacterium, constitutes a particularly attractive host due to its naturally high secretory capacity [19]. *B. subtilis* is a genetically highly amenable organism for which a large body of information and a variety of genetic tools are available. The laboratory strain *B. subtilis* 168 can develop natural competence [20], and therefore appears suitable for establishing robust and straightforward high-throughput transformation protocols.

These considerations lead us to develop a host-vector system for high-throughput cloning and inducible expression of heterologous genes in *B. subtilis*. An *E. coli – B. subtilis* shuttle vector which allows conditional expression of a gene of interest was converted to a Gateway-cloning compatible vector, and a *B. subtilis* host was genetically modified to make it compatible with a cell-based *in vitro* screen for immune modulation.

## Materials and Methods

### Bacterial Strains and Plasmids

The bacterial strains and plasmids used in this study are listed in **Table 1** **.** *E. coli* and *B. subtilis* were grown at 37°C in Luria-Betani (LB) broth with agitation or on the same medium solidified with 1.5% agar. Antibiotics were added where appropriate. For *B. subtilis*, erythromycin was used at 30 µg/mL, tetracycline at 10 µg/mL, spectinomycin at 50 µg/mL and chloramphenicol, kanamycin, and phleomycine at 5 µg/mL. For *E. coli*, spectinomycin was used at 100 µg/mL, ampicilin at 100 µg/mL and kanamycin at 25 µg/mL.

**Table 1:** Bacterial strains and plasmids used in this study.

Strains	Genotype	Relevant properties	Reference
***B. subtilis***			
168	*trp*C2		[45]
KA8AX	168 derivative; Δ*epr* Δ*wpr* Δ*mpr* Δ*nprB* Δ *bpr* Δ*nprE* Δ*vpr* Δ*aprE aprX*::spc	deficient in 9 proteases	[42]
VI7686	168 derivative; (*yvyD-yvzG-fliT-fliS-fliD-yvyC-hag*)::phleo	deficient in flagellin production	this work
VI7692	KA8AX derivative; (*yvyD-yvzG-fliT-fliS-fliD-yvyC-hag*)::phleo	deficient in 9 proteases and flagellin production	this work
VI7695	VI7692 derivative; (*tasA-sipW-yqxM-yqzG*)::cm (*epsA-O*)::tet	deficient in 9 proteases, flagellin and biofilm matrix production	this work
***E. coli***			
DB3.1	F^-^ *gyr*A462 *end*A1 Δ(*sr*1-*rec*A) *mcr*B *mrr hsd*S20(rB^-^, mB^-^) *sup*E44*ara*-14 *gal*K2 *lac*Y1 *pro*A2 *rps*L20(SmR) *xyl*-5 λ– *leu mtl*1	mutation in the gyrase allows the propagation of plasmids containing the *ccdB* gene	Invitrogen
One Shot®TOP10	F^-^ *mcr*A Δ(*mrr-hsd*RMS-*mcr*BC) φ80*lac*ZΔM15 Δ*lac*Χ74 *rec*A1 *ara*D139 Δ(*ara-leu*) 7697 *gal*U *gal*K *rps*L (StrR)*end*A1 *nup*G λ-	host for cloning with counter selection of *ccdB*	Invitrogen
TG1	F' [*tra*D36 *pro*AB+ *lac*Iq *lac*ZΔM15]*sup*E *thi*-1 Δ(*lac-pro*AB) Δ(*mcr*B-*hsd*SM)5, (rK^-^ mK^-^)	generation of plasmid multimers for use in transformation of *B.subtilis* competent cells	Lucigen
**Plasmids**			
pDONR223	Spc^r^ ccdB^+^	Gateway entry vector	Invitrogen
pDG148	Kan^r^ Amp^r^ Phl^r^	*E. coli* – *B. subtilis* shuttle expression vector	[31]
pBluescript SK+	Amp^r^	Multiple cloning site plasmid with pUC origin	Stratagene
pSK-A	Amp^r^ Cm^r^ ccdB^+^	pBluescript SK+ with RfA Gateway cassette	This work
pDG148-GW	Kan^r^ Amp^r^ Cm^r^ ccdB^+^	Gateway adapted pDG148	this work
pDGnuc	Kan^r^ Amp^r^	pDG148-GW derivative containing the staphylococcal nuclease gene (SA0746)	this work
pDGfliC-SEC	Kan^r^ Amp^r^	pDG148-GW derivative containing the *E. coli fliC* gene with signal peptide	this work
pDGfliC-CWA	Kan^r^ Amp^r^	pDG148-GW derivative containing the *E. coli fliC* gene with signal peptide and cell wall anchor	this work
pDGgfp	Kan^r^ Amp^r^	pDG148-GW derivative containing the *gfp-mut2* gene	this work

Deletion of genes from the *B. subtilis* chromosome was achieved through double crossing over recombination, replacing deleted sequences with antibiotic resistance markers. For this purpose, 1.5 kb genome fragments upstream and downstream of the interval to be deleted were amplified by PCR and joined to an antibiotic cassette in a joining PCR reaction [21]. The resulting PCR product was then used to transform *B. subtilis* competent cells according to [22] with appropriate antibiotic resistance selection. Chromosomal modifications were verified by PCR analysis.

**Figure 1:**
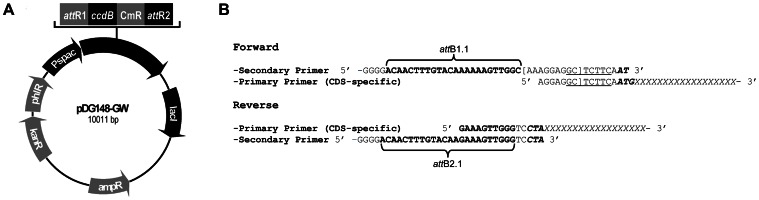
Schematic representation of the high-throughput cloning strategy. **A.** *B. subtilis* Gateway expression vector pDG148-GW with an inducible P*spac* promoter. lacI, lac repressor gene; AmpR, kanR, phlR, CmR, genes providing resistance to ampicilin, kanamycin, phleomycin, or chloramphenicol, respectively; ccdB, gene coding for the cytotoxic CcdB protein. **B.** Full-length coding sequences (CDS) are amplified by nested PCR using a set of CDS-specific (primary) primers and a set of universal (secondary) primers. The resulting PCR product contains the CDS preceded by a synthetic SD sequence (brackets) and a *Sap*I restriction site (underlined). *att*B1.1 and *att*B2.1 sites for site-specific recombination are indicated by braces; start and stop codons of the CDS, in italic-bold.

pDG148-GW was constructed using *E. coli* DB3.1 as a host for transformations. The Gateway reading frame cassette A (RfA, Invitrogen) was cloned in the *Eco*RV site of pBluescript SK+ (Stratagene). The orientation was verified by restriction with *Eco*RI and the plasmid with RfA orientated in the *Sac*I-*Kpn*I direction was selected and called pSK-A. The Gateway cassette RfA was then recovered from pSK-A after digestion with *Hind*III and *Xba*I, and ligated to pDG148 digested with the same enzymes to yield pDG148-GW (Fig. 1A).

Genes to be expressed in *B. subtilis* were amplified for cloning in pDG148-GW as schematically represented in Fig. 1B. Full-length coding sequences (CDS) were amplified by nested PCR using a set of gene specific primers and a set of universal primers in a 1∶4 ratio, and Phusion High Fidelity DNA polymerase (Finnzymes). CDS-specific primers were designed using PrimerDesigner1.0 (N. Pons, unpublished) so that the length of the CDS-specific primer sequences was at least 18 b and if necessary longer to obtain a theoretic annealing temperature of 55°C minimum (nearest-neighbor method [23]), and start and stop codons were systematically changed to ATG and TAG (amber), respectively. The Amber stop codon was used to permit C-terminal tagging of the protein in *B. subtilis* amber suppressor strains [24]. The resulting PCR products contain the CDS preceded by an AAAGGAGGC sequence to constitute a strong ribosome binding site (RBS) in *B. subtilis* (http://partsregistry.org). Between this sequence and the start codon, we included the TCTTCA sequence, thus creating a *Sap*I recognition sequence (GCTCTTC) (Fig. 1B). This restriction site allows the direct utilization of the pDONR-gene by restriction/ligation cloning methods. RBS and CDS are flanked by the site specific recombination sites *att*B1.1 and *att*B2.1 [25]. The amplification products were cloned in pDONR223 (Invitrogen) by site-specific recombination between the *att*B1.1 and *att*B2.1 sites and the *att*P1 and *att*P2 sites, respectively, present on the plasmid, *in vitro,* using BP clonase II (Invitrogen) according to the recommendations of the supplier, followed by transformation of OneShot^R^ TOP10 chemically competent *E. coli* (Invitrogen) with selection for spectinomycin (this *E. coli* strain allowing the counter selection of pDONR223 without insert). The resulting entry clones (25–50) were pooled and the plasmid inserts, now flanked by *att*L1 and *att*L2, were transferred to pDG148-GW by site-specific recombination between the *att*L1 and *att*L2 sites and the *att*R1 and *att*R2 sites, respectively, present on the latter plasmid, *in vitro*, using LR clonase II (Invitrogen), followed by transformation of *E. coli* TOP10 with selection for ampicilin. After each transformation, transformant colonies were pooled and plasmids extracted using a QIAprep Spin Miniprep Kit (Qiagen). The presence of inserts in pDONR223 and pDG148-GW was verified by digestion with *Bsr*GI and *Eco*RI (New England Biolabs), respectively, and PCR amplifications using the pDONR223-specific primers 5′-CCCAGTCACGACGTTGTAAAACG and 5′-GTAACATCAGAGATTTTGAGACAC. The plasmid pool obtained after LR recombination and transformation was used to transform chemically competent *E. coli* TG1, in order to generate multimeric plasmids suitable for transformation of competent *B. subtilis* [26]. Resulting clones were pooled, plasmids extracted and used to transform *B. subtilis* according to [22]. Three *B. subtilis* clones were verified by colony PCR using the vector-specific primers 5′-CGCACCCTGAAGAAGATTTA and 5′-GCCGACTCAAACATCAAATC. One clone was kept for further study.

The *gfp-mut2* gene, encoding a variant of green fluorescent protein (GFP) with improved photo-stability and brightness [27], was amplified from plasmid DNA (A. Chastanet, pers. comm.). Genes coding for secreted and cell-wall bound forms of the *E. coli* flagellin, obtained by cloning of the *fli*C coding sequence in pSEC [28] or pCWA [29], respectively (A. Barinov, personal comm.), were amplified from plasmid DNA. The nuclease encoding gene SA0746 was amplified from *Staphylococcus aureus* N315 genomic DNA.

### Heterologous Gene Expression in *B. subtilis* and Protein (activity) Detection

The expression of genes cloned in pDG148-GW in *B. subtilis* was induced by the addition of 2 mM Isopropyl β-D-1-thiogalactopyranoside (IPTG) to exponentially growing cultures (OD600 = 0.4), after which incubation was continued for 3 hours. GFP expression was observed under the microscope. Nuclease activity was measured in the culture supernatant using a spectrophotometric assay as described in [30]. Flagellin in bacterial cell wall fractions was detected by Western blotting: bacteria from 2 mL of a culture in which flagellin production had been induced were harvested by centrifugation, washed twice with ice-cold phosphate-buffered saline (PBS), resuspended in 100 µL of 10 mM Tris-HCl (pH 8) containing sucrose (1M), lysozyme (Sigma, 5 mg/mL), and a protease inhibitor cocktail (Roche), and incubated for 90 min at 37°C. The suspension was then centrifuged at 10,000 rpm for 18 min at 4°C, and the supernatant, corresponding to the cell-wall fraction, was subjected to SDS-polyacrylamide gel electrophoresis (12.5% acrylamide). Proteins were transferred to a nitrocellulose membrane, and probed with rabbit polyclonal antibodies to *E. coli* flagellin (Abcam, 1∶20,000 dilution). Interactions were identified using goat anti-rabbit IgG antibodies coupled to peroxidase (Sigma, 1∶10,000 dilution), and ECL detection (GE Healthcare) according to the recommendations of the supplier.

### Immune Modulation Assays

A human colon epithelial cell line HT-29 clone carrying a chromosomally located luciferase reporter gene under the control of an NF-κB dependent promoter (HT-29/NF-κB-luc-E, H. Blottière, pers. comm.) was used to evaluate immune modulation effects of *B. subtilis* strains and heterologously expressed flagellin. Alternatively, HEK-Blue™ TLR5 (Invivogen), a human embryonic kidney cell line, HEK293, that stably co-expresses human TLR5 and an NF-κB-inducible SEAP reporter gene, was used to quantify the response to flagellin. HT-29/NF-κB-luc-E cells were grown in RPMI 1640 (Lonza) and HEK- Blue TLR5 cells in DMEM (Lonza). Both culture media were supplemented with 2 mM L-glutamine, 50 IU/mL penicillin, 50 µg/mL streptomycin and 10% (or 20% for HEK-Blue TLR5) heat-inactivated fetal calf serum (FCS, Lonza). Cells were grown in a humidified 5% CO2 atmosphere (or 10% for HEK-Blue TLR5) at 37°C. The HT-29 reporter cell line was seeded at 5×10^4^ cells per well in 96-well plates (3917 assay plate, Costar, Cambridge, MA) and incubated for 48h in 100 µl of complete RPMI. The RPMI medium was refreshed before the immune modulation assay. For TLR5 stimulation 15×10^3^ HEK-Blue TLR5 cells were seeded per well. Induction of recombinant protein expression in *B. subtilis* was performed as described above. An amount of bacterial culture needed to prepare 100 µL of bacterial suspension with an OD_600nm_ of 0.5 was centrifuged, after which the bacterial pellet was washed and resuspended in 100 µL of RPMI or DMEM. 10 µL (5×10^6^ bacterial cells) were then added to the HT-29/NF-κB-luc-E (MOI = 25) or HEK-Blue TLR5 cells (MOI = 333) in a final volume of 100 µL. Alternatively, 10 µL of filtered bacterial culture supernatant (0.22 µm pore size filters (Millex GP, Milipore)) was used. Tumor necrosis factor alpha (TNF-α) (PeproTech, Rocky Hill, NJ)) and *Salmonella typhimurium* flagellin (FLA-ST) (Invivogen) where used where appropriate at final concentrations of 1 ng/mL and 10 ng/mL, respectively. For HT-29 cells, after 6 hours 50 µL of the RPMI medium was removed and luciferase reporter activity in the HT29 cells was measured using the One-Glow Luciferase Assay System (Promega) according to the manufacturer's instructions and a Tecan Spectrafluor Plus apparatus. Where appropriate, the removed medium was frozen at −80°C for further analysis, and 10 µL of this medium was used to quantify the amount of secreted IL8 by enzymed-linked immuno sorbent assay (ELISA; Biolegend, San Diego, CA) according to the manufacturer's recommendations. For HEK- Blue TLR5 cells, after 24 hours of stimulation, SEAP reporter activity was measured using the QUANTI-Blue™ SEAP detection reagent according to the manufacturer’s recommendations. For each assay, three wells were used and the mean result for the three wells considered as the assay result. All assays were performed in triplicate.

## Results

### High-throughput Gene Cloning and Expression in *Bacillus subtilis*

The development of a high-throughput system for the presentation of proteins from Gram-positive bacteria involved three criteria to meet the specific needs of functional metagenomics studies: the choice of a suitable host bacterium, a cloning strategy, and a cloning vector.

We chose to use a Gram-positive bacterium as a host, a prerequisite for the correct presentation of surface exposed and secreted proteins derived from Gram-positive bacteria, selected in metagenomic datasets. *B. subtilis* was chosen as the most exhaustively studied Gram-positive model bacterium, amenable to genetic modification to adapt it to the specific needs of a particular functional genomics project (see below).

For gene cloning we adopted the Gateway system [15] for site-specific recombination based cloning. We adapted the *E. coli* – *B. subtilis* shuttle vector pDG148 for use with this high-throughput cloning system by the introduction of the Gateway cassette. The resulting destination vector, pDG148-GW (Fig. 1A), allows the cloning of genes under the control of the IPTG-inducible P_spac_ promoter [31] for expression in *B. subtilis*. The subsequent steps of gene amplification, cloning and expression were established using the *gfp-mut2* gene as an example. The GFP coding sequence was amplified using a nested PCR approach (Fig. 1B), adding an *att*B1.1 site and a synthetic ribosome binding site to the 5′ end and an *att*B2.1 site to the 3′ end. The amplification product was cloned in pDONR223 (Invitrogen) by site-specific recombination between the *att*B1.1 and *att*B2.1 sites and the *att*P1 and *att*P2 sites, respectively, present on the plasmid. The plasmid insert from the resulting entry clone, now flanked by *att*L1 and *att*L2, was transferred to pDG148-GW by site-specific recombination between the *att*L1 and *att*L2 sites and the *att*R1 and *att*R2 sites, respectively, present on the latter plasmid, and the resulting plasmid was used to transform *E. coli* TG1 in order to generate multimeric plasmids, that were subsequently used to transform naturally competent *B. subtilis* (as transformation of competent *B. subtilis* with multimeric plasmids is 1000 fold more efficient than transformation with monomeric plasmids [26]). In *B. subtilis* 168, GFP expression could readily be induced by the addition of IPTG while no fluorescence was observed in the absence of IPTG (results not shown). Together, these results validate the functionality of the gene amplification, cloning and expression strategy.

### *B. subtilis* Host Adaptation for Functional Metagenomics Studies

**Figure 2:**
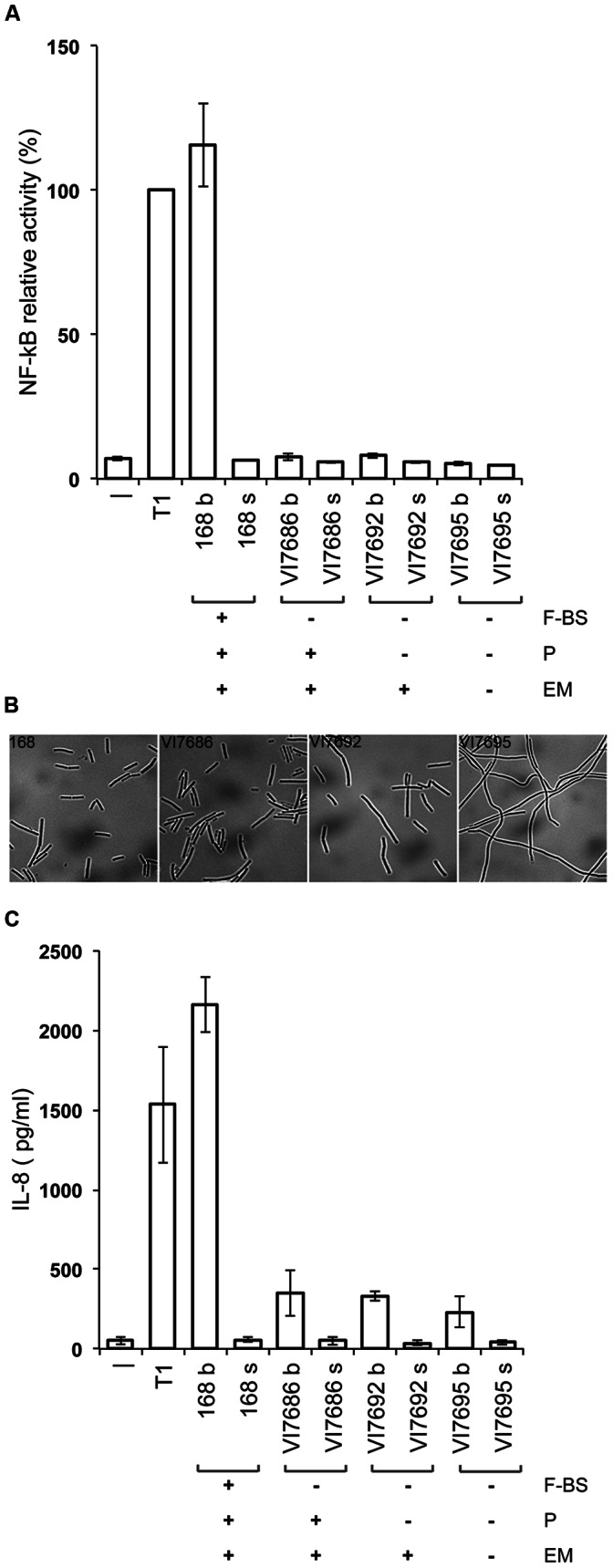
Properties of *B. subtilis wt* and mutant strains. **A.** Effect of *B. subtilis* strains on NF-κB activation. Bars indicate the relative activity of a luciferase reporter gene under the control of an NF-κB dependent promoter in an intestinal epithelial cell line (HT-29), *in vitro*. Bacterial strains are indicated on the abscissa. b, live bacteria from exponentially growing cultures; s, culture supernatant; -, HT-29 cells only; T1, TNF-α (1 ng/ml). Mean values of three independent experiments are presented. Error bars indicate the SEM. F-BS, presence (+) or absence (−) of *B. subtilis* flagellin gene; P, presence or absence of multiple *B. subtilis* protease genes; EM, presence or absence of extracellular matrix genes (*eps* and *tas*A). **B.** *B. subtilis* phenotypes as viewed by phase-contrast microscopy. Exponentially growing bacteria are shown, 100X magnification. **C.** Effect of *B. subtilis* strains on IL8 secretion. Bars indicate the concentration of secreted IL8 after incubation of HT-29 cells with the bacteria or bacterial culture supernatants indicated on the abscissa. Abbreviations as in Fig. 2A. The same bacterial cultures were used for the NF-κB activation assay shown in Fig. 2A and the IL8 secretion assay shown in Fig. 2C.

Protein presentation systems permit to explore the activities of individual heterologous proteins. In our case, we were interested in the use of a presentation system for the evaluation of potential immune modulation effects of heterologous proteins, in an assay where expression clones or their culture supernatants are brought into contact with human cell cultures. The assay we used monitors the activation of NF-κB, a key factor in the establishment of inflammatory responses [32], in an intestinal epithelial cell line [12]. *B. subtilis* 168 itself appeared to induce NF-κB activation in this assay (Fig. 2A), and we hypothesized that this effect could be ascribed to its flagellin. Flagellins of other bacteria have been described as potent NF-κB activators, acting through the activation of the TLR5– MyD88 signaling cascade [33], [34]. We therefore eliminated the flagellin and surrounding genes from the *B. subtilis* genome, and the resulting strain, VI7686, appeared to have lost its capacity to induce NF-κB activation (Fig. 2A). This result showed that an adapted *B. subtilis* strain could fulfill the requirement of background neutrality in the assay used.

A property of *B. subtilis* that is known to interfere with the efficient production of heterologous (extracellular) proteins is the production of a multitude of proteases. We therefore decided to use a 168-derived strain in which eight genes coding for extracellular proteases and one gene for an intracellular protease have been inactivated (strain KA8AX, [35]), and to eliminate the flagellin and surrounding genes from that background. The resulting strain, VI7692, was verified to behave neutral in the NF-κB activation assay (Fig. 2A), and its utility will be discussed below (performance of the host-vector system).

Two further deletions were made in strain VI7692 that could potentially improve the secretion and surface exposition of heterologous proteins: deletion of the *eps* operon and of the *tasA* gene that together have been reported to be involved in the production of an extracellular matrix [35]. The resulting strain, *B. subtilis* VI7695, still behaved neutral in the NF-κB activation assay (Fig. 2A), but showed an pronounced filamenting phenotype (Fig. 2B) where cells did not separate until stationary phase, and a viscous cell pellet after centrifugation. A similar phenotype was observed for a strain in which only the *eps* operon was deleted (results not shown). The different host adaptations did not significantly affect bacterial growth (results not shown).

In addition to their evaluation in the NF-κB activation assay, the *B. subtilis* strains described above were tested for their effect on the secretion of the chemokine IL8. IL8 plays an important role in the recruitment and activation of neutrophils at sites of infection or injury, and its expression is under the control of not only NF-κB, but also of several other signaling pathways [36]. The measurement of the expression of this chemotactic and inflammatory cytokine thus provides a complementary view on the capacity of *B. subtilis* to interact with gut epithelial cells. The results presented in Fig. 2C show that where *B. subtilis* 168 strongly induces IL8 secretion, the flagellin deletion mutants have lost most of this IL8 modulation capacity. When brought into contact with the latter bacteria, HT-29 cells secrete only about 15% of the quantity of IL8 they secrete when in contact with *B. subtilis* 168. The strains we present here may thus also be used in immune-modulation assays with an IL8 readout. The remaining low-level IL8 secretion may be due to the activation of other signaling pathways than the NFkB pathway by bacterial surface molecules, or by cellular stress [36].

### Performance of the Host – Vector System

**Figure 3:**
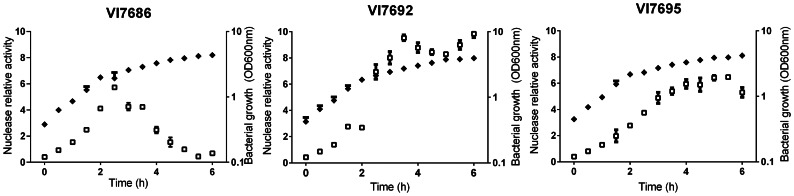
Activity of *S. aureus* nuclease in different *B. subtilis* backgrounds. Growth (OD_600 nm_) and nuclease relative activity in culture supernatants (scale 1–10) are presented for three different *B. subtilis* backgrounds. Filled diamonds, growth; open squares, nuclease relative activity. Time is time after the beginning of nuclease induction by addition of 2 mM IPTG. Mean values of three independent experiments are presented. Error bars indicate the SEM.

The effect of host adaptations on the production of heterologous secreted proteins was evaluated in two ways. First, using the strategy described above we cloned and expressed the *Staphylococcus aureus* nuclease (Nuc) encoding gene, and measured the nuclease activity in culture supernatants. The results presented in Fig. 3 show that in strain VI7686 (lacking flagellin), the nuclease activity reached a maximum about three hours after induction and subsequently declined to completely disappear after 5 h 30 of induction. Roughly the same activity curve was observed in the *wt* strain 168 (results not shown). In strain VI7692 (deficient in both flagellin and 9 proteases) nuclease activity reached, after about 3 hours, a 1.5 fold higher maximum than in the first two strains, and no decline was observed afterwards. The additional deletion of the *eps* and *tasA* genes in strain VI7695 did not improve nuclease production, but on the contrary retarded production while the maximum activity was comparable to the lower level observed in the *wt* strain 168. On the basis of this result and the earlier mentioned filamenting phenotype the VI7695 background was discarded for further experiments.

**Figure 4:**
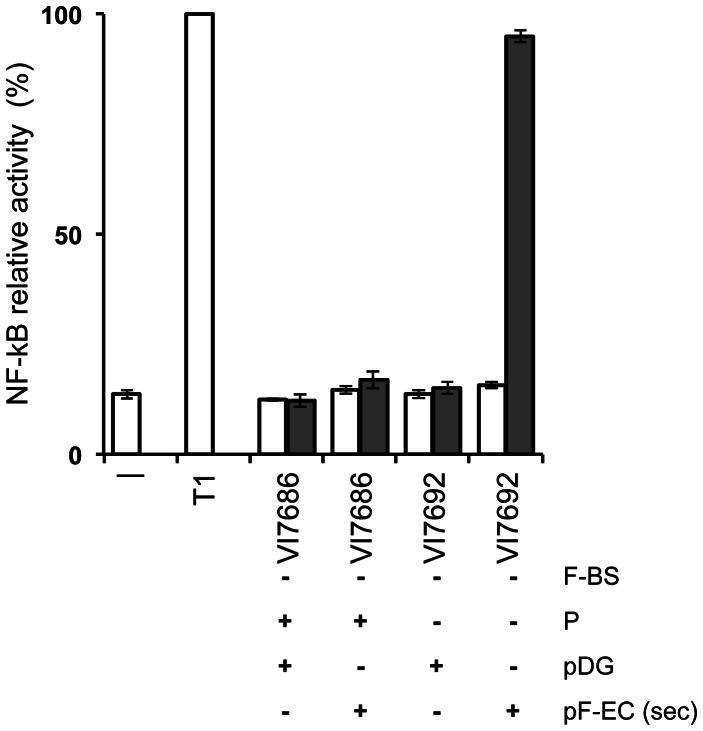
Immune modulation effect of secreted *E. coli* flagellin in different *B. subtilis* backgrounds. Bars indicate the relative activity of a luciferase reporter gene under the control of an NF-κB dependent promoter in an intestinal epithelial cell line (HT-29), *in vitro*. Bacterial backgrounds of which the culture supernatant was tested are indicated on the abscissa: grey bars, supernatants of bacterial cultures in which flagellin (if present) had been induced with IPTG; open bars, supernatants of bacterial cultures in which flagellin had not been induced. -, HT-29 cells only; T1, TNF-α (1 ng/ml). Mean values of three independent experiments are presented. Error bars indicate the SEM. F-BS, presence (+) or absence (−) of *B. subtilis* flagellin gene; P, presence or absence of multiple *B. subtilis* protease genes; pDG, presence or absence of pDG148 (empty vector); pF-EC, presence or absence of pDGfliC-SEC encoding a secreted form of *E. coli* flagellin.

We then expressed a secreted form of the *E. coli* flagellin, by cloning the *fliC* gene from *E. coli* DH10b fused in frame to the *usp*45 signal sequence from the Gram-positive bacterium *Lactococcus lactis* (A. Barinov, unpublished) in pDG148-GW. The resulting plasmid, pDGfliC-SEC, was used to transform *B. subtilis* VI7686 and VI7692 and, after induction of flagellin production, bacteria and culture supernatants were used to study NF-κB induction in human gut epithelial HT-29 cells, *in vitro*. While none of the washed bacterial cells induced NF-κB activation (results not shown), the supernatants of strain VI7692 (pDGfliC-SEC) in which flagellin production had been induced during 3 hours clearly induced NF-κB activation (Fig. 4). No activation of NF-κB was observed when flagellin production had not been induced. Surprisingly, when using the supernatant of induced cultures of strain VI7686 (pDGfliC-SEC), no activation at all was observed suggesting the complete degradation of the heterologous flagellin by the proteases produced by this strain. In order to verify that this result was characteristic for this host strain and not due to plasmid rearrangements, we isolated pDGfliC-SEC from this strain and used it to transform strain VI7692, and vice versa. Testing the resulting strains corroborated that the NF-κB activation results depended on the host background (results not shown). The different behavior of nuclease and flagellin in the VI7686 background may be explained by a differential sensitivity of nuclease and flagellin towards the various *B. subtilis* proteases.

The results of both nuclease and flagellin expression and the accompanying activity assays confirmed the functionality of the host – vector system presented in this work. Both also showed that the protease deficient background largely improved the production of heterologous secreted proteins, while the additional deletion of the *eps* and *tasA* operons did not result in further improvement or even seemed less optimal and caused a filamenting phenotype. The results of the flagellin expression assays in VI7692 demonstrate the utility of the system for protein presentation in screening for immune modulation properties.

### Presentation of Bacterial Surface Proteins

**Figure 5:**
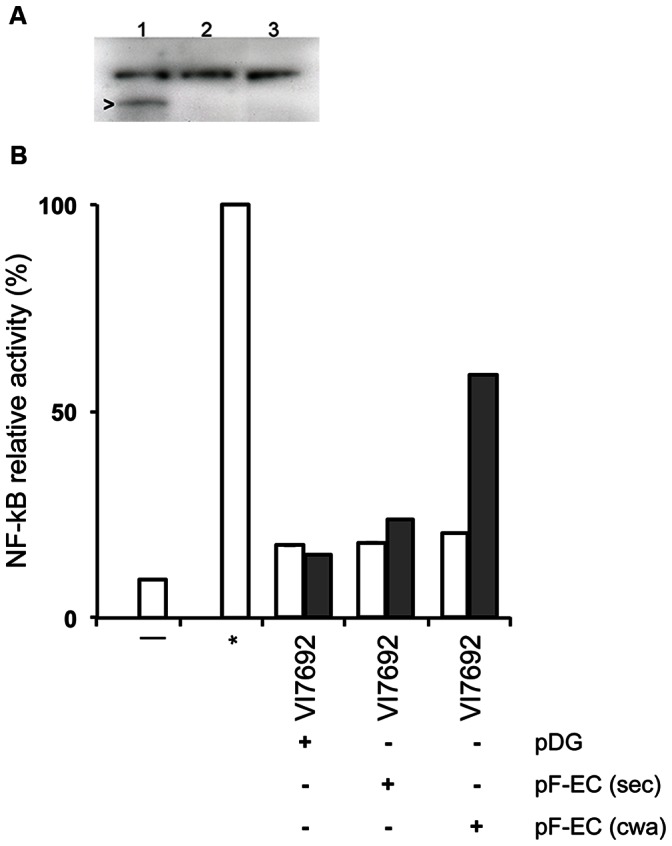
Presence and immune modulation effect of *E. coli* flagellin in *B. subtilis* cell wall fractions. **A.** *E. coli* flagellin in cell wall fractions of *B. subtilis* expressing the cell wall attached (lane 1) or the secreted (lane 2) form of flagellin, and a control expressing no flagellin (lane 3); Arrow points to the cell wall attached flagellin at the expected position of approximately 63 kDa. **B.** NF-κB activation capacity of washed *B. subtilis* VI7692 bacteria expressing a secreted (sec) or cell wall attached (cwa) form of *E. coli* flagellin. Grey bars, bacteria in which flagellin (if present) had been induced with IPTG; open bars, bacteria in which flagellin had not been induced. Bars indicate the relative activity of an SEAP reporter gene under the control of an NF-κB dependent promoter in HEK-Blue TLR5 cells, *in vitro*. -, HEK-Blue TLR cells only; *, *S. typhimurium* flagellin (10 ng/mL); pDG, presence (+) or absence (−) of pDG148 (empty vector); pF-EC(sec), presence or absence of pDGfliC-SEC encoding a secreted form of *E. coli* flagellin; pF-EC(cwa), presence or absence of pDGfliC-CWA encoding a cell wall attached form of *E. coli* flagellin. One representative experiment is shown out of two repetitions each performed in triplicate.

Bacterial surface proteins play an important role in bacteria-host interactions. Among the different classes of Gram-positive bacterial surface proteins (summarized in [37], [38]), a specific class, known as LP×TG proteins by the sequence motif they share, relies on a sortase for its covalent attachment to the bacterial cell wall [39]. *B. subtilis* contains two putative sortases [40] and the functionality of one of them has recently been demonstrated [41]. We chose to take the example of an LP×TG protein to validate the use of our system for the presentation of heterologous surface proteins and the subsequent evaluation of their immune modulation potential. For this purpose, we expressed an *E. coli* flagellin which, in addition to the N-terminal USP45 signal peptide, contained a C-terminal cell-wall anchoring LP×TG motif derived from the *Streptococcus pyogenes* M6 protein. The results of Western blotting using antibodies directed against the *E. coli* flagellin showed the presence of the flagellin in the bacterial cell wall fraction of the strain expressing the cell wall bound flagellin, while no flagellin was detected in the cell pellet of the strain expressing the secreted flagellin (Fig. 5A). We then studied the NF-κB activation potential of the bacterial cell pellet, using a human embryonic kidney HEK293 reporter cell line expressing the flagellin–specific Toll-like receptor TLR5 (HEK-Blue™ TLR5, Invivogen). While no NF-κB activation was observed with the resuspended bacteria from the control strain containing pDG148 or the strain expressing the secreted form of flagellin, the bacteria expressing the cell wall bound form of the flagellin clearly induced NF-κB activation (Fig. 5B). No NF-κB activation was observed in a HEK-Blue™ NF-κB reporter cell line without TLR5 (results not shown). Together, these results show that the heterologous LP×TG containing flagellin is expressed and at least partly retained at the bacterial cell surface, and responsible for NF-κB activation. They thus confirm the utility of our presentation system for the evaluation of the immune modulation potential of bacterial surface proteins.

## Discussion

The increasing amounts of sequence data and associated results of correlation studies generated in metagenomics projects allow the formulation of hypotheses concerning bacterial gene functions in relation to the ecosystem studied. The experimental validation of these hypotheses asks for new functional genomics approaches as the majority of bacterial species from the ecosystem-specific microbiota have never been cultured in the laboratory, and sequence data are mostly generated using Next Generation Sequencing technologies (NGS) that circumvent the need of cloning DNA fragments. As a result, specific bacterial species or clone libraries with identified insert content are most often not available for direct testing.

An alternative directed approach comes in sight as the increased predictive power from large datasets (enhanced *in silico* analysis capacities and accumulating *a priori* knowledge) will more and more allow to focus on relatively small, but still considerable, sets of genes (in the hundreds to thousands range) of potential interest, of which the separate cloning and screening can be envisaged using high-throughput methods. In this context, we realized that surface exposed and secreted proteins from Gram-positive bacteria constitute a category of proteins that may be hard to evaluate by cloning in *E. coli* due to the different architectures of the cell envelopes of Gram-positive and Gram-negative bacteria, and that a Gram-positive host would thus be preferable for these proteins. In addition, the screening for biological activity of these proteins requires that the expression host behaves neutral in the assay system used. We therefore set out to develop a compatible high-throughput protein presentation system that would, in our case, permit the evaluation of the immune modulation potential of individual proteins from these categories.

This objective implied the development of an integrated system comprising i) a suitable expression vector, compatible with high-throughput cloning strategies and allowing target gene expression in a Gram-positive host, and ii) a suitable host that would behave neutral in the envisaged immune modulation assay. For this purpose, we adapted the widely used *E. coli – B. subtilis* shuttle expression vector pDG148 for use with the Gateway high-throughput cloning system, and chose to use *B. subtilis* as the expression host. We also adapted a *B. subtilis* strain that had earlier been optimized for protein production through the deletion of multiple protease coding genes [42] to make it behave neutral in the immune modulation assay. It appeared that one chromosomal deletion encompassing the flagellin coding gene and six surrounding genes sufficed to eliminate the pro-inflammatory, NF-κB activating, behavior of the *B. subtilis* strain.

The expression and secretion of a heterologous flagellin capable of eliciting an immune modulation response in human HT-29 gut epithelial cells delivered the proof of principle of the utilization of the newly developed host-vector system for the presentation of secreted proteins. Moreover, we showed that the same flagellin when equipped with a C-terminal LP×TG motif was at least in part retained on the bacterial cell, and that the recombinant bacterial cells acquired the capacity to elicit a pro-inflammatory immune modulation response, thus demonstrating the utilization of our system for the functional presentation of bacterial cell surface proteins. This result contradicts earlier work with *B. subtilis* where the authors concluded that the *B. subtilis* sortase, which attaches the protein to the cell wall, does not recognize the canonical LP×TG motif but only the LPDTS motif [41], [43].

LP×TG proteins constitute only one type of surface proteins specific to Gram-positive bacteria. Another important but often overlooked class of proteins that can be surface exposed is constituted by proteins that contain one or more trans-membrane helices (TMHs) [37]. Due to the very different nature of the Gram-positive and Gram-negative cell-envelopes, surface exposition of this type of proteins from Gram-positive bacteria is expected to be correctly reconstituted only in a Gram-positive expression host.

In the course of our work, the construction of two other Gateway compatible expression vectors for use in a Gram-positive host was reported [44] confirming the need for Gram-positive vectors in this cloning system. These vectors are intended for the use in *L. lactis*, however, a host less suited for our purpose as its notorious acid production has a deleterious effect on the eukaryotic cells used in the immune modulation assay.

The results presented here represent the enabling prerequisites for a directed approach to the functional exploration of the Gram-positive fraction of the gut microbiota, notably regarding immune modulation. The host-vector system can easily be adapted to the study of other functions or other ecosystems through the adaptation of the expression host, to assure its neutrality in the activity screen that will be employed.
